# Natural APOBEC3C variants can elicit differential HIV-1 restriction activity

**DOI:** 10.1186/s12977-018-0459-5

**Published:** 2018-12-17

**Authors:** Brett D. Anderson, Terumasa Ikeda, Seyed Arad Moghadasi, Amber St. Martin, William L. Brown, Reuben S. Harris

**Affiliations:** 1 Department of Biochemistry, Molecular Biology and Biophysics, Masonic Cancer Center, Center for Genome Engineering, Institute for Molecular Virology, University of Minnesota, Minneapolis, MN USA; 2 Howard Hughes Medical Institute, University of Minnesota, 2231 6th St. S.E., Minneapolis, MN 55455 USA

**Keywords:** APOBEC3C, DNA cytosine deaminase, HIV-1, Human genetic variation, Innate immunity, Retrovirus restriction factor

## Abstract

**Background:**

The APOBEC3 (A3) family of DNA cytosine deaminases provides an innate barrier to infection by retroviruses including HIV-1. A total of five enzymes, A3C, A3D, A3F, A3G and A3H, are degraded by the viral accessory protein Vif and expressed at high levels in CD4+ T cells, the primary reservoir for HIV-1 replication in vivo. Apart from A3C, all of these enzymes mediate restriction of Vif-deficient HIV-1. However, a rare variant of human A3C (Ile188) was shown recently to restrict Vif-deficient HIV-1 in a 293T-based single cycle infection system. The potential activity of this naturally occurring A3C variant has yet to be characterized in a T cell-based spreading infection system. Here we employ a combination of Cas9/gRNA disruption and transient and stable protein expression to assess the roles of major Ser188 and minor Ile188 A3C variants in HIV-1 restriction in T cell lines.

**Results:**

Cas9-mediated mutation of endogenous A3C in the non-permissive CEM2n T cell line did not alter HIV-1 replication kinetics, and complementation with A3C-Ser188 or A3C-Ile188 was similarly aphenotypic. Stable expression of A3C-Ser188 in the permissive T cell line SupT11 also had little effect. However, stable expression of A3C-Ile188 in SupT11 cells inhibited Vif-deficient virus replication and inflicted G-to-A mutations.

**Conclusions:**

A3C-Ile188 is capable of inhibiting Vif-deficient HIV-1 replication in T cells. Although A3C is eclipsed by the dominant anti-viral activities of other A3s in non-permissive T cell lines and primary T lymphocytes, this enzyme may still be able to contribute to HIV-1 diversification in vivo. Our results highlight the functional redundancy in the human A3 family with regards to HIV-1 restriction and the need to consider naturally occurring variants.

## Background

Retroviruses, including the AIDS virus human immunodeficiency virus type-1 (HIV-1), must evade destruction by an extensive array of antiviral host proteins known as restriction factors [1–3]. The seven human APOBEC3 (A3) enzymes constitute an important arm of this innate network of restriction factors. These enzymes catalyze the deamination of cytosine to uracil in single-stranded (ss) DNA substrates, and APOBEC3D (A3D), APOBEC3F (A3F), APOBEC3G (A3G) and APOBEC3H (A3H) are known to contribute to HIV-1 restriction (reviewed by [4, 5]). These four enzymes package into budding virions and, following virus entry into a new target cell, catalyze the formation of uracil lesions in reverse-transcription intermediates. These uracils template the incorporation of adenines during synthesis of the viral genomic strand, resulting in G-to-A mutations.

HIV-1 and related lentiviruses avoid lethal levels of A3 induced mutation through the virion infectivity factor (Vif), which is a small viral protein required for virus infectivity in most cell types. Vif functions as a molecular adapter to recruit A3 enzymes to a host ubiquitin ligase comprised of CBF-β, CUL5, RBX2, ELOB, and ELOC for polyubiquitination and subsequent degradation by the proteasome [6–8]. HIV-1 Vif effectively triggers the degradation of human A3D, A3F, A3G and A3H, which would otherwise restrict virus replication [9]. Through CBF-β recruitment, Vif also downregulates the transcriptional activity of these *A3* genes and thereby contributes to a permissive environment for virus replication [10].

Curiously, although human APOBEC3C (A3C) elicits little Vif-deficient HIV-1 restriction activity, Vif efficiently targets it for degradation [9, 11–13]. Human A3C also is highly expressed in the primary cellular reservoir for HIV-1 replication, CD4+ T cells, and is upregulated upon HIV-1 infection similar to the other restrictive A3 proteins [9]. Moreover, human A3C also potently restricts a strain of simian immunodeficiency virus (SIV) isolated from African Green Monkeys (SIVagm) in the absence of Vif in 293T-based single cycle infection experiments [11, 14]. These observations combine to indicate that human A3C is a *bona fide* retrovirus restriction enzyme and, further, that HIV-1 (or a SIV precursor from chimpanzees) may have recently evolved a Vif-independent mechanism for evading restriction by this enzyme (since little restriction is observed in the absence of Vif).

The *A3* gene family exhibits significant variation within the human population [15]. First, a deletion spanning the entire *A3B* coding sequence occurs at 37% frequency worldwide with clear geographic biases [16, 17]. Second, *A3H* has at least 7 distinct haplotypes that exhibit drastically different expression levels and HIV-1 restriction phenotypes [17–21]. The most common A3H haplotype (hap I) is a poorly expressed protein with little to no Vif-deficient HIV-1 restriction activity, and it is found at a 48% frequency worldwide and implicated in cancer mutagenesis [17–22]. In contrast, the next most common A3H haplotype (hap II) is well expressed and shows strong Vif-deficient HIV-1 restriction activity [17–22]. Third, two recent reports have characterized a rare A3C-Ile188 variant (~ 2% global allele frequency) with enhanced restriction activity against Vif-deficient HIV-1 in a 293T based single cycle infection model in comparison to the predominant A3C-Ser188 enzyme [11, 23]. Biochemical studies have indicated that the enhanced restriction activity of A3C-Ile188 may be due to increased enzyme processivity for cytosine deamination and/or to an increased propensity to homodimerize [11, 23].

The impact of the rare A3C-Ile188 enzyme on HIV-1 restriction in T lymphocytes has yet to be studied. Here, using a combination of Cas9/gRNA and stable expression approaches in T cell lines, we show that neither genetic disruption of *A3C* in the non-permissive CEM2n T cell line, nor complementation with natural A3C variants is sufficient to alter HIV-1 replication kinetics in the presence or absence of Vif. However, stable expression of A3C-Ile188 can slow Vif-deficient virus replication and inflict G-to-A mutations in the permissive SupT11 T cell line in the absence of other A3 proteins. Taken together, these findings confirm prior reports identifying the rare Ile188 allele as a more restrictive form of A3C. Our studies underscore the possibility that this naturally occurring A3C variation may influence HIV-1 genetic variation and pathogenesis in vivo.

## Results

### Immunoblot comparisons of epitope-tagged and untagged A3C variants

**Fig. 1:**
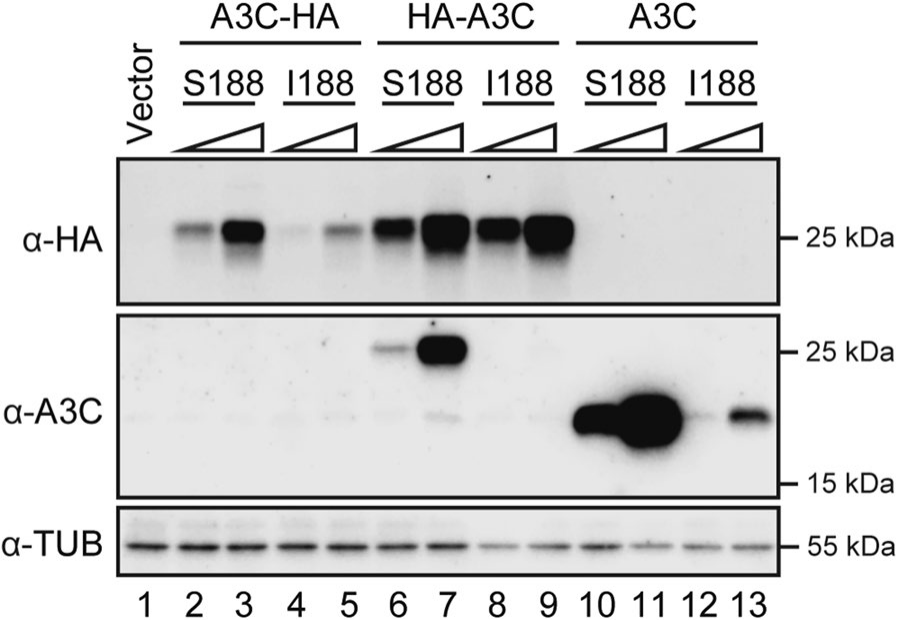
Expression of epitope-tagged and untagged A3C derivative constructs in 293T cells. Immunoblots of 293T cells transfected with 100 ng or 400 ng of C-terminally tagged (A3C-HA), N-terminally tagged (HA-A3C), or untagged A3C variants using either an anti-HA or anti-A3C antibody for detection. Tubulin was used as a loading control

The first study comparing A3C-Ser188 and A3C-Ile188 used a C-terminal HA tag and reported lower expression levels of the C-terminally tagged Ile188 variant [15]. A more recent report using C-terminally HA-tagged A3C variants in a different vector showed no significant expression difference [11]. Our results using different C-terminally triple-HA-tagged variants mirror the original report with the Ile188 variant expressing at lower levels (Fig. 1, top blot, lanes 2/3 vs 4/5). Another recent study used N-terminally HA-tagged variants and found no expression difference [23]. Our results with different N-terminally triple-HA-tagged A3C variants confirm this observation (Fig. 1, top blot, lanes 6/7 vs 8/9). We postulate that the observed instability of the Ile188 variant may be due to an interaction with the C-terminal HA-epitope tag in some, but not all, vectors (i.e., a potential epitope tag-associated artifact).

In an attempt to resolve this issue, we tested all available commercial anti-A3C antibodies in immunoblot experiments and found only one reagent that reproducibly detected both transfected and endogenous A3C (Proteintech #10591-1-AP; see validation in Cas9/gRNA disruption studies below and “Methods” for a list of reagents that failed). This polyclonal antibody was raised against full-length A3C, and it enabled confirmation of a subset of the anti-epitope blots above and yielded an additional new consideration. First, it showed that the N-terminally HA-tagged A3C-Ser188 construct expresses much better than the reciprocal C-terminally HA-tagged construct (Fig. 1, middle blot, lanes 6/7 vs 2/3). Second, it strongly detected HA-A3C-Ser188 but only weakly the Ile188 variant (Fig. 1, middle blot, lanes 6/7 vs 8/9). This expression difference was particularly clear for untagged A3C proteins (Fig. 1, middle blot, lanes 10/11 vs 12/13). Because these proteins only differ by one amino acid, these data strongly indicate that the dominant antibody in the polyclonal mixture recognizes an epitope that requires Ser188 and is somehow compromised by Ile188. Consistent with this interpretation, a different single amino acid substitution, Ser188 to Leu, also renders A3C undetectable in immunoblot experiments with this antibody (data not shown). These epitope tag and immunoblot limitations impose significant constraints on studies with A3C.

### Single cycle infectivity results using 293T cells

**Fig. 2:**
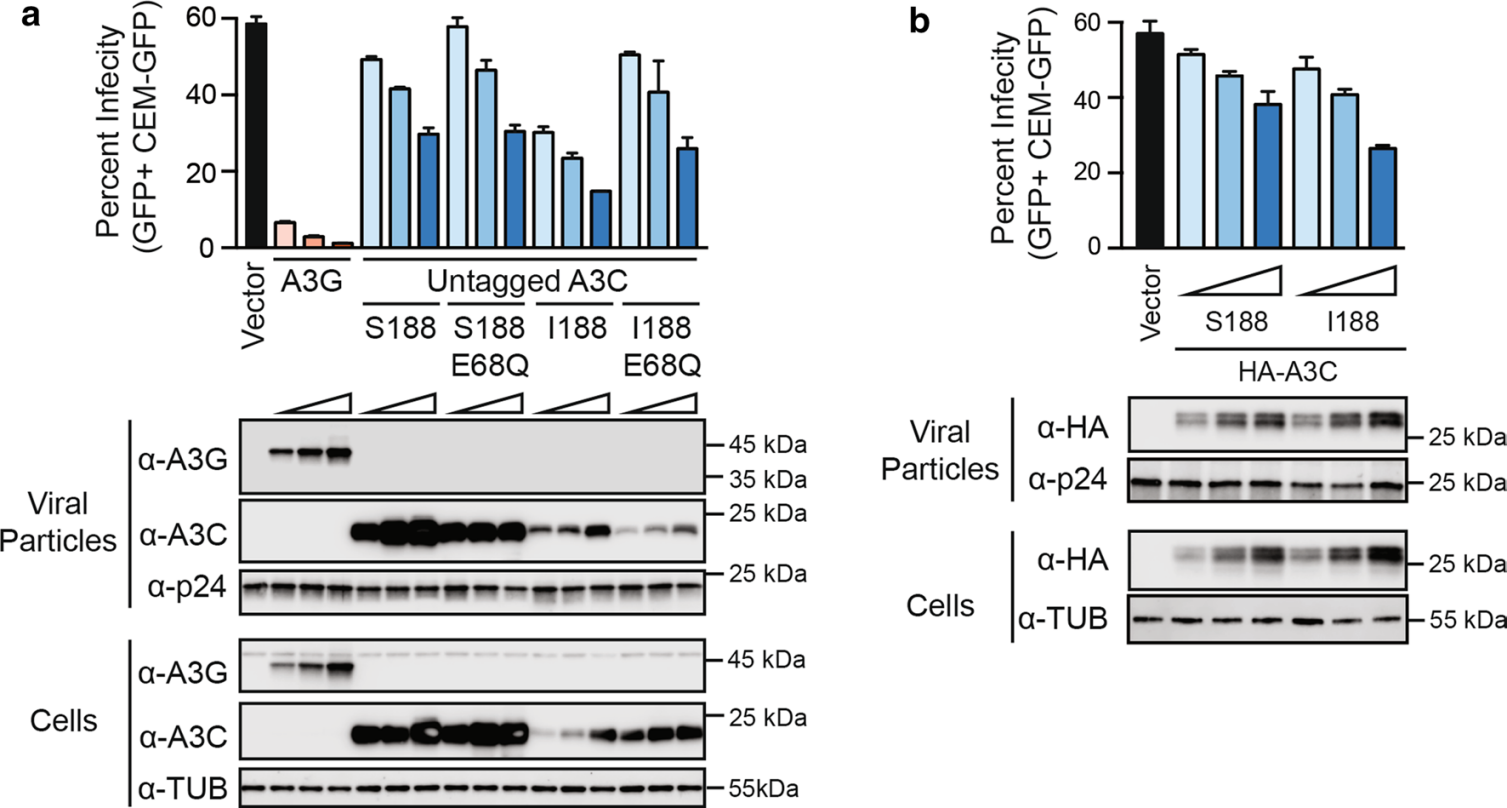
A3C-Ile188 exhibits enhanced HIV-1 restriction activity in 293T cells. **a** Single cycle infectivity data for Vif-deficient HIV-1 viruses produced in the presence of untagged A3C-S188, A3C-I188, or catalytic mutant derivatives (E68Q). Immunoblots are shown below for viral particles (anti-A3G, anti-A3C, and anti-p24) and producer cells (anti-A3G, anti-A3C, and anti-tubulin). **b** Single cycle infectivity data for Vif-deficient HIV-1 viruses produced in the presence of N-terminally HA-tagged A3C-S188 or A3C-I188. Immunoblots are shown below for viral particles (anti-HA and anti-p24) and producer cells (anti-HA and anti-tubulin). All single cycle experiments were repeated at least 3 times, with representative infectivity data (mean ± SD) and immunoblots shown for one experiment

Because untagged, and to a lesser degree N-terminally HA-tagged, A3C-Ser188 displays visibly higher expression levels than C-terminally HA-tagged derivatives, untagged and N-terminally HA-tagged constructs were used for functional studies from here onward. In agreement with prior reports [11, 23], untagged A3C-Ser188 caused a modest twofold reduction in the infectivity of Vif-deficient HIV-1 in the 293T-based single cycle infection system (Fig. 2a). A comparison of this A3C-Ser188 construct and a Glu68-to-Gln derivative indicated that effect is largely independent of the conserved catalytic glutamate. In contrast, A3C-Ile188 caused a larger fourfold infectivity decline that partly required deaminase activity (Fig. 2a). Parallel reactions included A3G as a strong positive control and an empty expression vector as a negative control (Fig. 2a). In comparison, similar restriction activities were observed using N-terminally tagged A3C Ser188 and Ile188 expression constructs with a trend toward greater restriction by the Ile188 protein at higher expression levels (Fig. 2b). The untagged and N-terminally tagged constructs were not analyzed in the same experiments in parallel so head-to-head comparisons of restriction activity are not possible. The most important point from these single cycle infectivity experiments is that the Ile188 enzyme is approximately twofold more restrictive than the Ser188 enzyme.

### A3C disruption and variant complementation has little effect on HIV-1 replication kinetics in non-permissive CEM2n cells

**Fig. 3:**
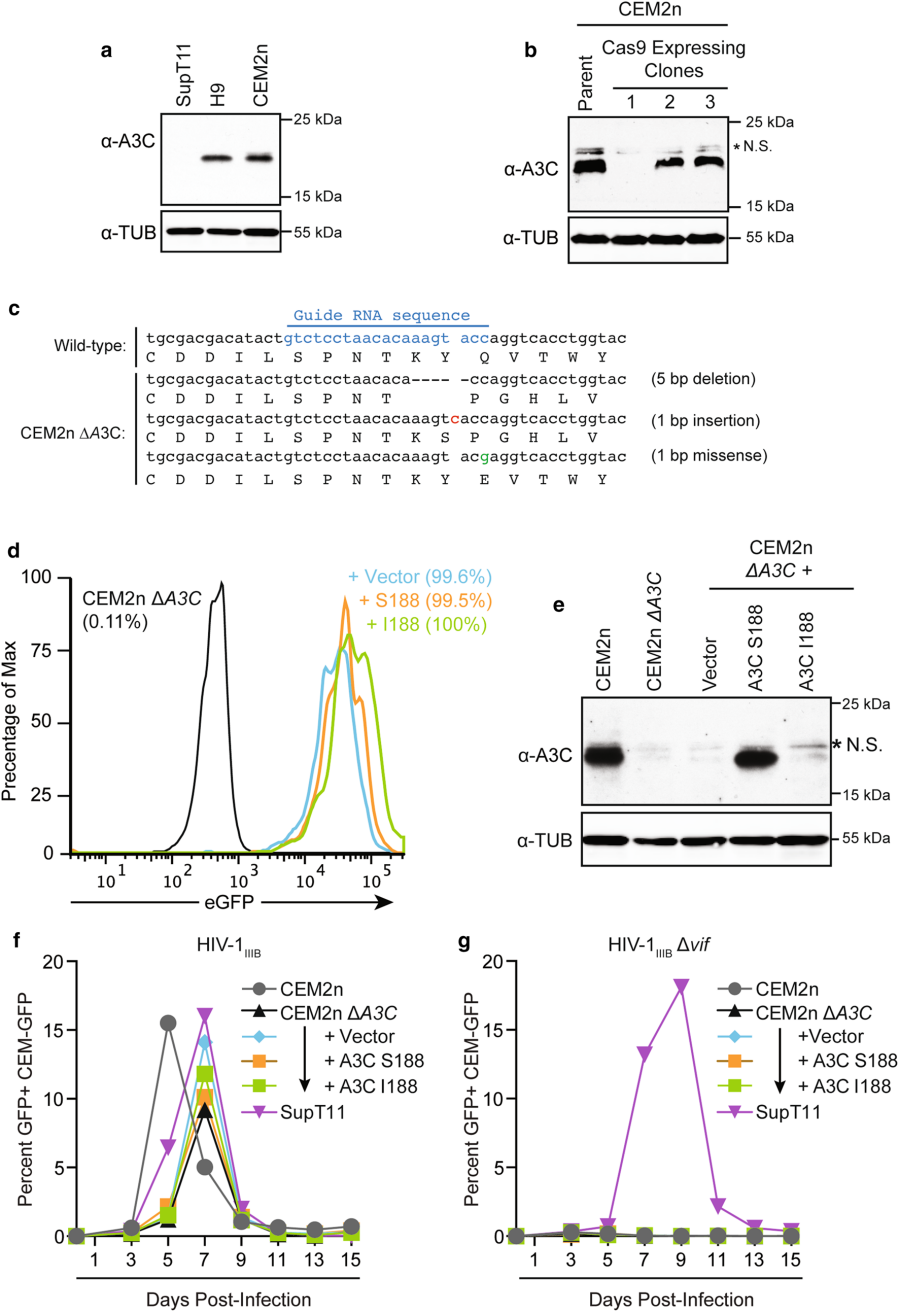
HIV-1 replication phenotypes following *A3C* disruption and variant complementation in non-permissive CEM2n cells. **a** Immunoblots of endogenous A3C in SupT11, H9, and CEM2n cells. Tubulin was used as a loading control. **b** Immunoblots of endogenous A3C in CEM2n clones following targeted disruption of *A3C* exon 3 by Cas9/gRNA complexes. Tubulin was used as a loading control. **c** *A3C* exon 3 sequences from parental CEM2n and an A3C-disrupted clone (CEM2n ∆*A3C*). **d** Flow cytometry plots for CEM2n Δ*A3C* cell pools 72 h post-transduction with GFP-reporter complementation vectors. The percentage of GFP+ cells is indicated for each population. **e** Immunoblots of A3C in the parental CEM2n line, CEM2n Δ*A3C*, and complemented CEM2n Δ*A3C* derivatives. Tubulin was used as a loading control. **f**, **g** Spreading infection kinetics of Vif-proficient and Vif-deficient HIV-1 (initial MOI = 0.02) in CEM2n, CEM2n Δ*A3C*, and the indicated complemented conditions. SupT11 cells are included as a control permissive cell type. Virus infectivity was determined by infection of CEM-GFP with culture supernatants followed 48 h later by quantification with flow cytometry. Each spreading infection experiment was performed 3 times and representative data are shown

Little is known about the role of endogenous A3C in HIV-1 restriction in T cells. Endogenous A3C is detected readily in non-permissive H9 and CEM2n T cell lines, but not in the permissive SupT11 T cell line using the commercial anti-A3C antibody described above (Fig. 3a). Cas9/gRNA-mediated genome editing was used to disrupt endogenous *A3C* in CEM2n [24]. The parental CEM2n line was transduced with a vector expressing Cas9 and an *A3C*-specific guide RNA, and limiting dilution was used to isolate clones harboring no detectable A3C protein by immunoblot (e.g., clone 1 in Fig. 3b). Genomic DNA sequencing of the gRNA-targeted exon 3 region of *A3C* revealed multiple mutant alleles and, importantly, no wild-type sequences (Fig. 3c). The presence of 3 distinct alleles indicates that the original near-diploid cell line CEM2n [24] may have increased ploidy during culture or duplicated the *A3C* region of the genome. Regardless, A3C-disrupted clones showed no overt phenotypes such as morphology or growth rates in comparison to non-targeted sister clones (data not shown).

Next, a complementation experiment was set-up by transducing the *A3C* mutant clone described above with viruses expressing A3C-Ser188, A3C-Ile188, or an empty vector as a negative control. Each construct also expressed eGFP to control for transduction efficiency (and to help mitigate the fact that the untagged A3C-Ile188 variant cannot be detected with available antibodies, as shown in Fig. 1). In all instances, near complete transduction efficiency was confirmed by GFP+ flow cytometry (Fig. 3d). Moreover, immunoblotting showed that complemented A3C-Ser188 expression levels are similar to endogenous levels normally found in the parental CEM2n line (Fig. 3e). As explained above, protein-level expression of A3C-Ile188 could not be measured accurately due to Ser188 likely being part of the main epitope recognized by the antibody. Nevertheless, these two variants are expected to express at similar levels because the vectors are otherwise isogenic, the Ser188 and Ile188 variants express similarly with N-terminal tags, and the eGFP levels of the complemented pools are almost indistinguishable (and results below show a phenotype with the same A3C-Ile188 construct).

To address the role of endogenous A3C (Ser188/Ser188) and to compare complemented A3C-Ser188 and A3C-Ile188 variants in parallel in HIV-1 spreading infections, transduced cell pools were infected with HIV-1_IIIB_ or a Vif-deficient derivative and virus replication was monitored over time. Neither the Cas9/gRNA-mediated disruption of endogenous A3C nor complementation with either natural A3C variant altered the kinetics of Vif-proficient virus replication with all CEM2n ΔA3C derivatives showing peak infectivity at 7 days post-infection (Fig. 3f). In contrast, CEM2n ΔA3C derivatives remained fully non-permissive to Vif-deficient virus replication presumably due to expression of other endogenous restrictive A3 enzymes including A3D, A3F, A3G and A3H [9, 24] (Fig. 3g). Vif-deficient virus replication in the permissive SupT11 cell line confirmed infectivity of the viral stock (Fig. 3f, g). Taken together, these results demonstrate that A3C disruption is not sufficient to render CEM2n cells detectably permissive to Vif-deficient HIV-1 replication, and that complementation with A3C-Ser188 or A3C-Ile188 is unable to alter the kinetics of virus replication in the presence of Vif, potentially because both variants are targeted for degradation by Vif at similar efficiencies [11].

### Stable expression of A3C-Ser188 and A3C-Ile188 in SupT11 cells restricts Vif-deficient HIV-1 replication and inflicts G-to-A mutations

**Fig. 4:**
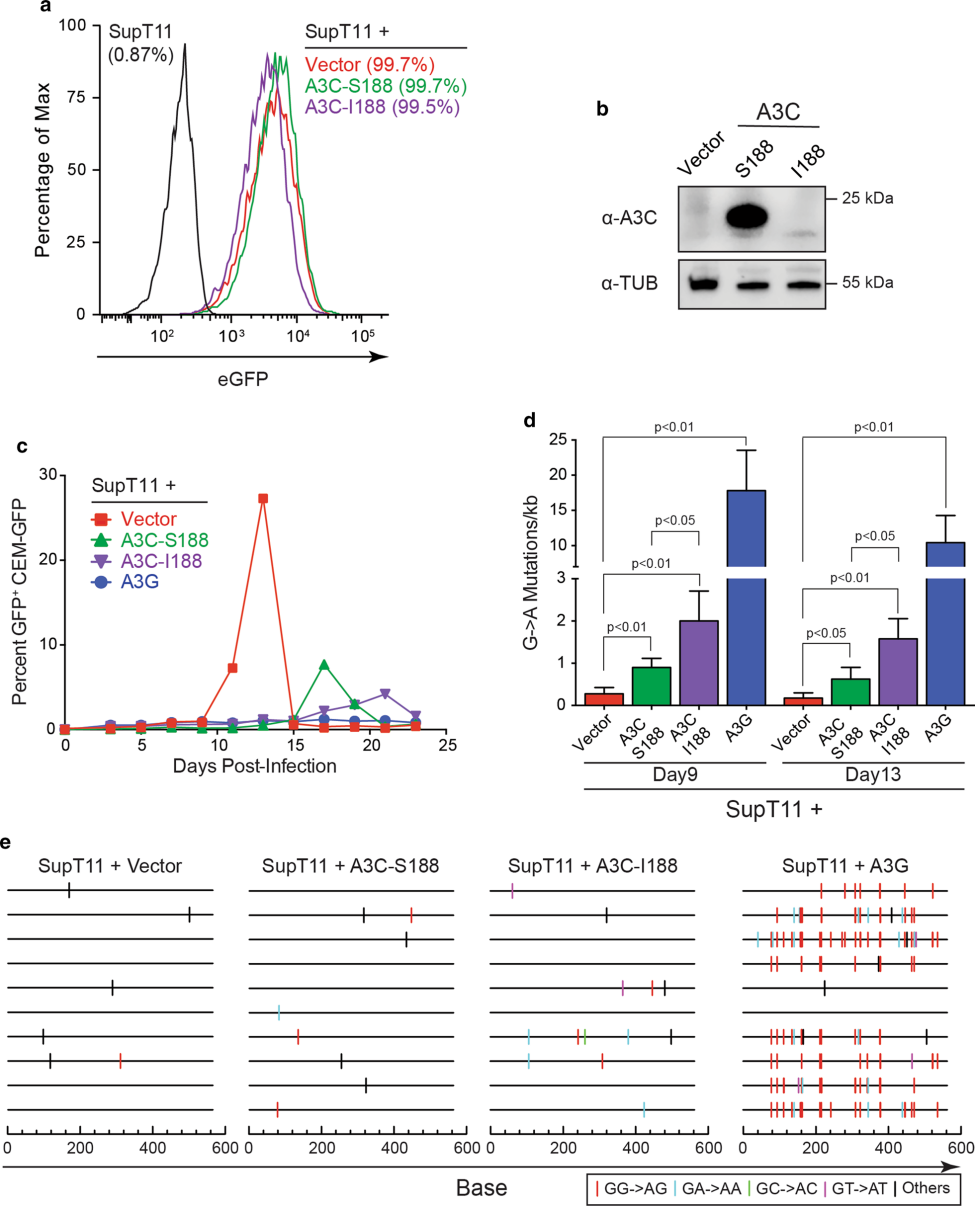
Stable Expression of both A3C-Ile188 and A3C-Ser188 in SupT11 cells provides a partial block to Vif-deficient HIV-1 replication. **a** Flow cytometry plots for SupT11 cells 72 h after transduction with the indicated GFP-marked complementation vectors. The percentage of GFP+ cells is indicated for each condition. **b** Immunoblots of A3C in SupT11 cells transduced with constructs expressing A3C-Ser188, A3C-Ile188, or an empty control vector. Tubulin was used as a loading control. **c** Representative spreading infection data for Vif-deficient HIV-1 (MOI = 0.01) in SupT11 cells expressing A3C-Ser188, A3C-Ile188, or an empty control vector. Virus infectivity was determined by infection of CEM-GFP with culture supernatants followed 48 h later by quantification with flow cytometry. These data are from one of four biologically independent experiments. **d** Average G-to-A mutation loads for each condition. Error bars show ± SD of 4 independent experiments. Statistical comparisons were done using Student’s *t* test. *p* values above each panel are in comparison to vector control or A3C-Ser188. **e** Mutation data for each condition

The phenotypically redundant antiviral activities of A3D, A3F, A3G, and A3H make it challenging to study A3C activity in non-permissive T cell lines such as CEM2n. Therefore, permissive, non-A3 expressing T cell lines such as SupT11 provide robust systems for comparing the antiviral activities of single DNA deaminases and mutant derivatives [9, 20, 25–27]. Prior to knowledge of A3C-Ile188, we reported that A3C-Ser188 is unable to restrict Vif-deficient HIV-1 when expressed stably in this system [9]. These studies were additionally limited by the utilization of a C-terminal triple-HA epitope tag, which based on results above is detrimental to A3C expression (Fig. 1). Therefore, the intrinsic restriction activities of untagged A3C-Ser188 and A3C-Ile188 were compared following stable transduction and expression in SupT11 cells. As above, efficient transduction was confirmed by GFP+ flow cytometry for all constructs (Fig. 4a) and by immunoblotting for A3C-Ser188 (Fig. 4b).

The resulting SupT11 pools were infected with Vif-deficient HIV-1 (MOI = 0.01) and virus replication kinetics were monitored over time. Both A3C-Ser188 and A3C-Ile188 suppressed virus replication, but the latter variant imposed a reproducibly but not significantly greater delay (Fig. 4c). Moreover, in addition to imposing replication delays, the spreading infection peaks were lower than those in the vector control condition (28 ± 14, 12 ± 10, and 9 ± 6% GFP-positive reporter cells for vector, A3C-Ser188, and A3C-Ile188 conditions, respectively; n = 4 biologically independent experiments).

To ask whether A3C inflicts G-to-A mutations in HIV-1 genomic sequences, high-fidelity PCR was used to recover proviral DNA from the HIV-1 infected SupT11 derivatives after 9 and 13 days of spreading infection. A minimum of 10 independent 564 bp *pol* region DNA amplicons were cloned and sequenced for each condition. These analyses revealed that both A3C variants caused significant increases in overall G-to-A mutation loads (twofold for Ser188 and fourfold for Ile188; Fig. 4d, e). In both instances, the underlying mutation spectra showed evidence for both 5′-GA-to-AA and 5′-GG-to-AG mutations due to cDNA strand 5′-TC-to-TU and 5′-CC-to-CU deamination events, respectively (Fig. 4e). These proviral DNA sequencing analyses showed that both A3C-Ser188 and A3C-Ile188 have the capacity to inflict G-to-A mutations in viral DNA during a spreading infection in a T cell model system. However, in direct comparison to A3G, much lower frequencies of G-to-A mutation are observed suggesting some of the antiviral activity of A3C may also be due to a deamination-independent mechanism (supported by data in Fig. 2a).

## Discussion

The A3 DNA cytosine deaminase family imposes a significant barrier to productive HIV-1 infection, especially for viruses lacking Vif. Prior studies converged on A3D, A3F, A3G and A3H as the HIV-1 restrictive A3 repertoire in CD4+ T cells (e.g., [9, 20, 28, 29]; reviewed by [2, 4, 30]). Our current studies indicate that A3C-Ile188 has the potential to contribute to HIV-1 restriction in human T cells. Our studies confirm prior 293T-based single cycle results [11, 23] and, importantly, extend this conclusion to a relevant model T cell line SupT11. Importantly, our studies show that both the common (Ser188) and the rare (Ile188) A3C variants are capable of inflicting G-to-A mutations during spreading infection in T lymphocytes with the rare variant inflicting a twofold higher mutation frequency. This increase in G-to-A mutations may be explained by an increased capacity to dimerize and to function in a processive manner [23]. This result raises the possibility that both variants may contribute to HIV-1 diversity. However, additional work will be needed to extend these findings to primary T lymphocytes, for instance, through an analysis of endogenous A3C variants following Cas9/gRNA-mediated deletion of A3D/F/G/H *ex vivo*. Such studies would also be facilitated by an anti-A3C monoclonal antibody (yet to be developed to our knowledge) that binds equally well to both naturally occurring A3C-Ser188 and A3C-Ile188 variants.

The seven gene human *A3* locus is variable in the human population, consistent with an overall function of the encoded enzymes in innate immunity and previously documented evidence for positive selection [11, 31–34]. Known variations include complete inactivation of *A3* family members (29.5 kb *A3B* deletion [16] and A3H-∆Asn15 [18]), hypomorphic alleles (A3H-G105 [18]), and hypermorphic alleles such as the A3C-Ile188 variant (prior work [11, 23] and this study). Several other coding and non-coding variants have been documented but, thus far, their functional relevance is less clear.

A3C is conserved in primates and phylogenetic comparisons show evidence for positive selection, including multiple amino acid substitutions within the predicted Vif-binding interface [11, 13, 35]. This suggests an ongoing conflict between ancestral lentiviruses with Vif and ancestral A3C enzymes. Consistent with this idea, cross species comparisons have shown restriction of SIVagm by human A3C [11, 14]. Taken together with the fact that HIV-1 Vif still degrades A3C and HIV-1 infection induces *A3C* expression [9, 14, 35–37], it is possible that A3C-Ser188 and A3C-Ile188 still impose some (albeit low) selective pressure on present day HIV-1 (or SIV in recent evolutionary history) [9]. Alternatively, A3D and/or A3F are still so similar to A3C that molecular mimicry within the Vif binding surface may be sufficient to maintain Vif function against A3C.

A3C amino acid 188 is located in the C-terminal helix, which is physically separated from the Vif binding interface [13]. However, phylogenetic comparisons for this region are interesting. Within the human A3 repertoire, most enzymes have Ile at the analogous structural position suggesting a need for greater restriction activity [11]. Sequence comparisons across divergent primate species indicate that Ile188 is the ancestral primate residue, occurring in divergent primate species including the orangutan, gibbon, siamang, and all sequenced Old World monkeys [11]. In contrast, Ser188 is found in humans and other hominids including chimpanzees, gorillas, and bonobos. Curiously, chimpanzee and gorilla A3C proteins have a HIV-1 restriction activity that is comparable to human A3C-Ile188, despite harboring Ser at the analogous amino acid position, suggesting that additional amino acid differences between hominid species may play a role in defining intrinsic restriction activity [23]. Biochemical studies of these enzymes have revealed a correlation between self-dimerization of A3C in solution and HIV-1 restriction activity (observed for human A3C-Ile188, chimp A3C, and gorilla A3C, but not human A3C-Ser188) [23]. The biochemical characterization of additional primate A3C proteins will be needed to test the broad relevance of dimerization in A3C-dependent restriction of primate lentiviruses, though these findings strongly suggest that Ile188 is not the only determinant for enhanced restriction activity.

The overall contribution of A3C-Ile188 to HIV-1 replication in vivo remains unclear at this time, though relative comparisons in single cycle infection experiments suggest that its antiviral activity will pale in comparison to the potent restriction activity of A3G (e.g., Fig. 2a). Nevertheless, studies here and recent reports [11, 23] on A3C combine to reveal an additional example of genetic variation within the human A3 repertoire that may influence HIV-1 evolvability and pathogenesis in the primary cellular reservoir, CD4+ T cells. Future studies leveraging large patient cohorts of known *A3C* genotypes, with due attention to other variations within the locus, may be able to address this possibility.

## Conclusions

We show that the rare human A3C-Ile188 variant exhibits enhanced restriction activity against Vif-deficient HIV-1 in a 293T-based single cycle infection system, providing independent verification of prior work [11, 23]. Using Cas9-mediated gene disruption in non-permissive CEM2n T cells, we found that endogenous A3C is not required for Vif-deficient HIV-1 restriction, most likely due to the dominant restriction activities of A3D, A3F, A3G and A3H. Complementation of A3C-disrupted CEM2n cells also had no effect on virus replication kinetics. However, stable expression of A3C-Ile188 can convert the normally permissive SupT11 T cells to a less permissive phenotype with respect to Vif-deficient virus replication. Vif-deficient viruses also accumulate G-to-A mutations from A3C-Ile188, as well as from A3C-Ser188, under these spreading infection conditions. Taken together, these findings support a role for A3C in HIV-1 restriction and G-to-A mutation in T cells, and highlight an additional level of variation within the human *A3* gene locus that may effect HIV-1 adaptation and pathogenesis in vivo.

## Methods

### Cell lines

SupT11 [9], SupT11-A3G [38], CEM2n [24] and derivative cell lines were maintained in RPMI (Hyclone) supplemented with 10% FBS (Thermo Fisher) and 1% penicillin/streptomycin. 293T cells were maintained in DMEM (Hyclone) supplemented with 10% FBS (Gibco) and 1% penicillin/streptomycin. 293T cells were transfected with TransIT LTI (Mirus) according to the manufacture’s protocol. All experiments were harvested 48 h post-transfection.

### A3C expression plasmids

The C-terminally triple-HA tagged A3C construct in the pcDNA3.1(+) backbone has been described [9]. The N-terminally triple-HA tagged A3C constructs were ligated into the pcDNA3.1(+) backbone with a triple-HA epitope sequence between the *Nhe*I and *Hind*III cloning sites. Untagged A3C constructs were assembled similarly into an epitope tag-free pcDNA3.1(+) backbone. Untagged A3C variants were subcloned into the CSU6-IDR2 IRES-eGFP lentiviral expression vector (previously described [10] but modified here to express eGFP instead of dsRed) for stable transduction experiments in CEM2n and SupT11 cells.

### Immunoblots

Cell pellets were lysed directly in 2.5x Laemmli sample buffer, separated by 12.5% SDS-PAGE, and transferred to PVDF-FL membranes (Millipore). Membranes were blocked in 4% milk in PBS and incubated with primary antibodies diluted in 4% milk in PBS supplemented with 0.1% Tween20. Secondary antibodies were diluted in 4% milk in PBS supplemented with 0.1% Tween20 and 0.01% SDS. Membranes were imaged on a LI-COR Odyssey instrument. Primary antibodies used in these studies were rabbit anti-A3C (Proteintech 10591-1-AP), rabbit anti-A3G (NIH AIDS Reagent Program 10201 courtesy of J. Lingappa), rabbit anti-HA (Cell signaling C29F4), mouse anti-Tubulin (Sigma T5168), mouse anti-HIV-1 p24/CA (NIH AIDS Reagent Program 3537 courtesy of B. Chesebro and K. Wehrly), and mouse anti-HIV-1 Vif (NIH AIDS Reagent Program 6459 courtesy of M. Malim). Secondary antibodies employed were IRdye 800CW goat anti-rabbit (LI-COR 827-08365) and Alexa Fluor 680 goat anti-mouse (Molecular Probes A-21057), except when detecting the anti-A3C antibody, which was probed with an anti-Rb HRP-conjugated secondary antibody (Bio Rad 1706515) and visualized using Super Signal West Femto Maximum Sensitivity Substrate (ThermoFisher Scientific). The following A3C antibodies failed to detect overexpressed N-terminally tagged A3C variants by immunoblotting: rabbit anti-A3C (Abcam ab181356), rabbit anti-A3C (Abcam ab209560), rabbit anti-A3C (LSBio LS-B14538), and mouse anti-A3C (Sigma SAB1403204).

### HIV-1 single-cycle infectivity experiments

293T-based HIV-1 single cycle infectivity assays were performed by co-transfecting 293T cells with an HIV-1_IIIB_ A200C *vif*-deficient molecular clone derivative (pIIIB Δ*vif*) along with the indicated APOBEC3 expression construct or an empty vector control [39, 40]. 48 h later, cell free supernatants containing progeny viruses were used to infect CEM-GFP reporter cells. Reporter cell infectivity was assayed 48 h post infection by flow cytometry using a Becton Dickinson FacsCanto II instrument.

### *A3C* disruption by Cas9/gRNA

A gRNA sequence homologous to 5′-GGG GCT CCG CAG CCT GAG TC-3′ in *A3C* exon 3 was generated using the CRISPR Design Tool (Massachusetts Institute of Technology) and cloned into the lentiCRISPRv1 vector (Addgene #49535) according to the accompanying Zhang lab protocol [41]. This targeting construct was generated by annealing oligos 5′-ACA CCG TCT CCT AAC ACA AAG TAC CG-3′ and 5′-AAA ACG GTA CTT TGT GTT AGG AGA CG-3′ and ligating the resulting dsDNA fragment into *Bsm*BI-digested lentiCRISPRv1. 293T cells were transfected with the lentiCRISPRv1 targeting construct along with pΔ-NRF (HIV-1 *gag*, *pol*, *rev*, *tat* genes) and pMDG (VSV-G) expression vectors and, 48 h later, virus-containing supernatants were filtered (0.45 µm) and concentrated by centrifugation (22,000×*g*, 2 h, 10 °C). Viral pellets were resuspended in complete RPMI and incubated with cells for 48 h before being placed under drug selection (1 µg/mL puromycin). Clones were isolated by limiting dilution of the drug resistant cell pool, and A3C expression was analyzed by immunoblotting lysates from individual clones.

### A3C complementation

CSU6-IDR2 IRES-eGFP vectors encoding untagged and N-terminally HA-tagged A3C variants were co-transfected into 293T cells with p∆-NRF (HIV-1 *gag*-*pol*-*rev*-*tat*) and pMDG (VSV-G). 48 h post transfection, culture supernatants were filtered (0.45 µm) and concentrated by centrifugation (22,000×*g*, 2 h, 10 °C). Viral pellets were resuspended in complete RPMI and used to directly infect SupT11 and CEM2n target cells. Transduced cell populations were assayed for GFP expression by flow cytometry and A3C expression was measured by immunoblotting 72 h post-transduction.

### HIV-1 spreading infections

HIV-1 spreading infections were performed as described [9]. Briefly, replication-competent HIV-1 stocks were produced by transfecting 293T cells with HIV-1_IIIB_ A200C wild-type (pIIIB) or *vif*-deficient (pIIIB Δ*vif*) molecular clone derivatives [39, 40]. 48 h post-transfection, culture supernatants were filtered (0.45 µm), and viral titers were determined by serial titration on CEM-GFP reporter cells. CEM2n and SupT11 derivative cell lines were infected accordingly, and virus replication was monitored every other day (beginning on day 3 post-infection) by infecting CEM-GFP reporter cells with culture supernatants. Infected CEM-GFP cells were fixed at 48 h post-infection and assayed for GFP expression by flow cytometry.

### Proviral DNA G-to-A mutation analyses

SupT11 cells at day 9 and 13 of spreading infections were harvested and genomic DNA (including proviral DNA) was prepared (Puregene) for sequencing analysis as described [42]. The viral *pol* region was amplified by nested PCR with RSH4196/4197 (5′-TCC ART ATT TRC CAT AAA RAA AAA-3′ and 5′-TTY AGA TTT TTA AAT GGY TYT TGA-3′) as outer primers (876 bp) and RSH4205/4206 (5′-AAT ATT CCA RTR TAR CAT RAC AAA AAT-3′ and 5′-AAT GGY TYT TGA TAA ATT TGA TAT GT-3′) as inner primers (564 bp) and then the amplified products were subjected to pJET cloning and Sanger DNA sequencing (GENEWIZ). Sequences were aligned using Sequencher (Gene Codes Corporation) and analyzed for hypermutation by the Los Alamos Hypermut algorithm [43].
